# Screening and Identification of Cadmium-Tolerant, Plant Growth-Promoting Rhizobacteria Strain KM25, and Its Effects on the Growth of Soybean and Endophytic Bacterial Community in Roots

**DOI:** 10.3390/plants14152343

**Published:** 2025-07-29

**Authors:** Jing Zhang, Enjing Yi, Yuping Jiang, Xuemei Li, Lanlan Wang, Yuzhu Dong, Fangxu Xu, Cuimei Yu, Lianju Ma

**Affiliations:** 1 College of Life Science, Shenyang Normal University, Shenyang 110034, China; 18141740592@163.com (J.Z.); 17614256922@163.com (E.Y.); 18341576116@163.com (Y.J.); lxmls132@163.com (X.L.); wangqi5387402006@aliyun.com (L.W.); 18811657916@163.com (Y.D.); xufangxu321@163.com (F.X.); 2 College of Agronomy, Shenyang Agricultural University, Shenyang 110161, China

**Keywords:** PGPR, Cd, endophytic bacterial community, semi-wild soybeans

## Abstract

Cadmium (Cd) is a highly toxic heavy metal that can greatly affect crops and pose a threat to food security. Plant growth-promoting rhizobacteria (PGPR) are capable of alleviating the harm of Cd to crops. In this research, a Cd-tolerant PGPR strain was isolated and screened from the root nodules of semi-wild soybeans. The strain was identified as *Pseudomonas* sp. strain KM25 by 16S rRNA. Strain KM25 has strong Cd tolerance and can produce indole-3-acetic acid (IAA) and siderophores, dissolve organic and inorganic phosphorus, and has 1-aminocyclopropane-1-carboxylate (ACC) deaminase activity. Under Cd stress, all growth indicators of soybean seedlings were significantly inhibited. After inoculation with strain KM25, the heavy metal stress of soybeans was effectively alleviated. Compared with the non-inoculated group, its shoot height, shoot and root dry weight, fresh weight, and chlorophyll content were significantly increased. Strain KM25 increased the superoxide dismutase (SOD), peroxidase (POD), and catalase (CAT) activities of soybean seedlings, reduced the malondialdehyde (MDA) content, increased the Cd content in the roots of soybeans, and decreased the Cd content in the shoot parts. In addition, inoculation treatment can affect the community structure of endophytic bacteria in the roots of soybeans under Cd stress, increasing the relative abundance of Proteobacteria, Bacteroidetes, *Sphingomonas*, *Rhizobium*, and *Pseudomonas*. This study demonstrates that strain KM25 is capable of significantly reducing the adverse effects of Cd on soybean plants while enhancing their growth.

## 1. Introduction

Cadmium (Cd), a primary pollutant in soil contamination, is characterized by its harmful effects, high toxicity, ease of accumulation, extensive mobility, and resistance to degradation [1]. The presence of Cd not only poses potential risks to ecosystems but also adversely affects crop growth, ultimately endangering human health through bioaccumulation in the food chain [2]. Soybean (*Glycine max*) ranks as one of the world’s most economically significant crops globally, being rich in high-quality protein, abundant carbohydrates, and various minerals [3]. However, as a result of mining and smelting operations, as well as the application of pesticides and fertilizers, soybean is heavily affected by Cd contamination in the soil [4]. When Cd enters the soybean, it decreases its respiration and photosynthesis, increases oxidative stress, and disrupts the balance of nutrient uptake, primary metabolism, and hormones, which ultimately leads to a decrease in crop yield and quality [5]. The prolonged intake of soybeans contaminated with Cd can pose risks to human health, leading to a range of health problems. Currently, numerous conventional physical and chemical approaches have been utilized to address Cd contamination in agriculture. Nevertheless, these approaches tend to be costly, time-consuming, and prone to causing secondary contamination, thus limiting their feasibility for large-scale implementation [6]. Hence, it is necessary to identify efficient, economical, and eco-friendly technology to address soil Cd pollution and alleviate the adverse effects of Cd on soybeans, which will help to safeguard human health and maintain agroecosystem stability.

Plant growth-promoting rhizobacteria (PGPR) refer to a category of advantageous microorganisms that colonize the plant rhizosphere. These microorganisms are capable of stimulating plant growth directly and indirectly, while also enhancing plants’ tolerance to heavy metals [7]. Genera such as *Pseudomonas*, *Bacillus*, *Rhizobium*, *Enterobacter*, and *Azotobacter* can produce plant hormones, including indole-3-acetic acid (IAA), cytokinin (CK), abscisic acid (ABA), and gibberellin (GA), which enhance root length, surface area, and root hair density. These physiological alterations enhance root development and optimize water and nutrient absorption in plants [8]. In addition, bacteria such as *Pseudomonas* and *Bacillus* are capable of secreting organic acids (such as citric acid, oxalic acid, and malic acid), which transform insoluble phosphorus, potassium, and other soil nutrients into plant-available forms, thus enhancing nutrient accessibility in the soil [9]. They can also synthesize siderophores to chelate Fe^3+^ in the soil, facilitating the uptake of iron, zinc, and other trace elements by plants [10]. Additionally, bacteria such as *Pseudomonas*, *Bacillus*, and *Acinetobacter* generate 1-aminocyclopropane-1-carboxylate (ACC) deaminase to decompose the precursor of ethylene in plants, mitigating the damage caused by adverse conditions [11].

Meanwhile, when plants are under Cd stress, PGPR can also regulate the physiological and metabolic responses of plants, such as enhancing the antioxidant defense system and photosynthesis of plants, and regulate the absorption and transport of heavy metals by plants to resist heavy metal stress and reduce the toxic effects on plants [12,13]. PGPR can induce the synthesis of metal-chelating proteins in plant roots (phytochelatins and metallothioneins), which reduce Cd^2+^ toxicity by chelating cytoplasmic Cd^2+^ [14]. Additionally, PGPR regulates the expression of heavy metal transporter genes (HMA2, NRAMP1), thereby inhibiting Cd^2+^ translocation from roots to shoots [15]. For example, Mitra et al. isolated *Enterobacter* sp. S2 from Cd-contaminated soil, which exhibits multiple plant growth-promoting (PGP) traits. Its inoculation increased rice seedling biomass under Cd stress, alleviated Cd-induced oxidative stress, and reduced Cd accumulation in seedlings [16]. The inoculation strain *Enterobacter aerogenes* sp. K6 enhanced the superoxide dismutase (SOD) and catalase (CAT) activities of rice seedlings under Cd stress, reduced the malondialdehyde (MDA) content, alleviated the oxidative stress of rice seedlings, and improved the Cd tolerance of rice seedlings [17]. The plant microbiome encompasses various microbial communities attached to the surface and inside plants, including symbiotic microorganisms, mutualistic microorganisms, and pathogenic bacteria that are harmful. They play a crucial role in plant growth and development by expanding the plant genome and enhancing its metabolic capabilities [18]. Światczak et al. demonstrated that *Pseudomonas sivasensis* 2RO45 and *Bacillus paralicheniformis* 2R5, isolated from the rhizosphere of rape, significantly promote rape growth, enhance the abundance of beneficial microorganisms in the rhizosphere, boost the metabolic activity of the rhizosphere microbial community, and modify the functional diversity of the rhizosphere microbiome [19]. The inoculation of strain *Pseudomonas* E3 can significantly induce changes in the rhizosphere microbial community structure of *Solanum nigrum*, specifically manifested as an increase in the relative abundance of Proteobacteria and Actinobacteria, while the relative abundance of Firmicutes decreases [20]. Thus, given their remarkable growth-promoting traits, PGPR can serve as an effective tool to stimulate plant growth, reduce the adverse effects of Cd toxicity, and improve plants’ resilience to heavy metals.

At present, a variety of PGPR with Cd tolerance have been identified, mainly including *Pseudomonas*, *Bacillus*, *Azotobacter*, *Burkholderia*, *Arthrobacter*, *Klebsiella*, *Enterobacterium*, *Bradyrhizobium*, and *Flavobacterium* [21,22,23,24]. The genus *Pseudomonas* consists of rod-shaped, Gram-negative bacteria that exhibit plant growth-promoting abilities and strong resistance to pathogens and heavy metals [25]. *Pseudomonas citronellolis* KM594397 is capable of producing IAA, solubilizing phosphate, exhibiting strong tolerance to arsenic (As^5+^), and exerting a significant promotional effect on chickpea growth under As^5+^ stress [26]. Inoculation with *Pseudomonas aeruginosa* promoted the root length, stem length, and fresh weight of tomato seedlings under Cd stress, increased photosynthetic pigments, and alleviated Cd toxicity [27]. Rojas-Sánchez reported that inoculation with *Pseudomonas fluorescens* strain UM270 altered the endophytic microbiome in maize roots [28]. Dobrzyński et al. found that colonization of rapeseed roots by *Pseudomonas* sp. G31 increased the abundance of Firmicutes in the soil [29]. However, studies on how Pseudomonas affects the endophytic bacterial community structure in plant roots are relatively limited, with existing research mostly focusing on crops such as maize and rapeseed. Systematic studies on soybean roots under Cd stress are particularly scarce.

Semi-wild soybean (*Glycine gracilis*) is a transitional type in the evolution from wild soybean (*Glycine soja*) to cultivated soybean, presenting a unique growth state. It has rapid development and reproduction, strong resistance, high yield in the same year, rich protein content, and low production cost, playing an indispensable and important role in modern agriculture. Therefore, the objectives of this study were as follows: (1) to isolate and screen a Cd-resistant PGPR strain (designated KM25) from root nodules of semi-wild soybeans and identify its species via 16S rRNA sequence analysis; (2) to evaluate the potential of strain KM25 in alleviating Cd stress in soybean seedlings; (3) to clarify how inoculation with KM25 affects the structure of endophytic bacterial communities in the rhizosphere of soybean seedlings using high-throughput sequencing.

## 2. Results

### 2.1. Isolation of Cadmium-Tolerant Bacterial Strains from Semi-Wild Soybean Root Nodules and Identification of Strain KM25

**Figure 1:**
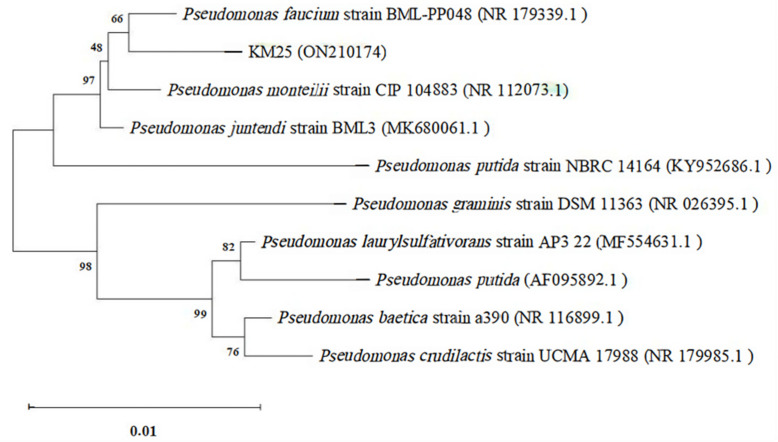
The phylogenetic tree was constructed based on 16S rRNA gene sequences using the neighbor-joining method. Numbers on branches indicate bootstrap support values from 1000 replications. Evolutionary distances were computed using the Kimura 2-parameter model, and the scale bar represents 0.01 substitutions per nucleotide position. Strain KM25, isolated in this study (GenBank accession number: ON210174), is marked with sequences of other reference strains retrieved from GenBank, with their accession numbers shown in parentheses.

A total of 12 bacterial strains were isolated and purified from the root nodules of semi-wild soybeans, and subsequently designated as KM01, KM04, KM06, KM10, KM14, KM15, KM18, KM19, KM25, KM32, KM34, and KM38. The growth conditions of each strain under different concentrations of Cd^2+^ stress are shown in (Table 1). As shown in Table 1, strain KM25 exhibits a high tolerance to Cd^2+^. Therefore, strain KM25 was determined as the preferred Cd-tolerant strain. The strain was characterized by using molecular biology techniques and was initially classified as *Pseudomonas* sp. KM25 (Figure 1). The strain with the highest similarity to strain KM25 was *Pseudomonas faucium* strain BML-PP048 (NR 179339.1), exhibiting a sequence identity of 99.72% and a coverage of 99%. The sequence of strain KM25 has been deposited in the GenBank database, with the corresponding gene sequence accession number ON210174.

### 2.2. PGP Traits

To further characterize the plant growth-promoting potential of strain KM25, its key PGP traits—including phosphorus solubilization, siderophore production, IAA secretion, and ACC deaminase activity—were systematically evaluated. The strain KM25 had a stronger ability to dissolve inorganic phosphorus than organic phosphorus, with the amount of dissolved inorganic phosphorus reaching 20.81 μg·mL^−1^ and the amount of dissolved organic phosphorus being 6.59 μg·mL^−1^. The strain KM25 had a strong ability to produce iron carriers, with the relative content of siderophores being 52.32%. Simultaneously, strain KM25 secreted IAA at a concentration of 5.16 μg·mL^−1^ and exhibited ACC deaminase activity of 12.13 U·mg^−1^.

### 2.3. Biomass and Chlorophyll Content

**Figure 2:**
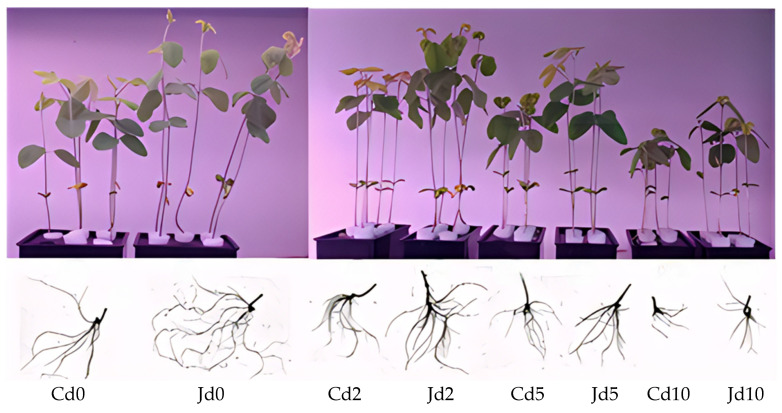
The growth conditions of soybean seedlings at different Cd concentrations; Cd0, Cd2, Cd5, and Cd10 indicate that the concentrations of CdCl_2_ in the seedling culture solution are 0, 2, 5, and 10 μg/mL, respectively. Jd0, Jd2, Jd5, and Jd10 indicate that 0, 2, 5, and 10 μg/mL of CdCl_2_, as well as 5% of KM25, were added to the culture solution, respectively.

Given the plant growth-promoting traits exhibited by strain KM25 as identified earlier, we further investigated its potential to alleviate Cd-induced growth inhibition in soybean seedlings. Under Cd stress, the growth of soybean seedlings was inhibited to different extents. However, inoculation with strain KM25 markedly reduced the inhibitory effects of Cd on soybean seedling growth (Figure 2). Under varying concentrations of Cd stress, compared to J− seedlings (uninoculated with strain KM25), the growth parameters of J+ seedlings (inoculated with strain KM25) in both the shoot and root parts (with the exception of root length) were significantly enhanced (Table 2). In the absence of Cd stress, the shoot height, shoot fresh weight, shoot dry weight, root fresh weight, root dry weight, and chlorophyll content of J+ seedlings increased by 25.79%, 8.01%, 41.34%, 6.04%, 18.7%, and 10.91%, respectively, compared to the control. However, when the Cd concentration was 2 μg/mL and 5 μg/mL, compared with J− seedlings, the root length of J+ seedlings decreased by 25.3% and 13.1%, respectively, but their chlorophyll content increased by 6.52% and 4.89%, respectively.

### 2.4. Antioxidant Systems and Lipid Peroxidation

**Figure 3:**
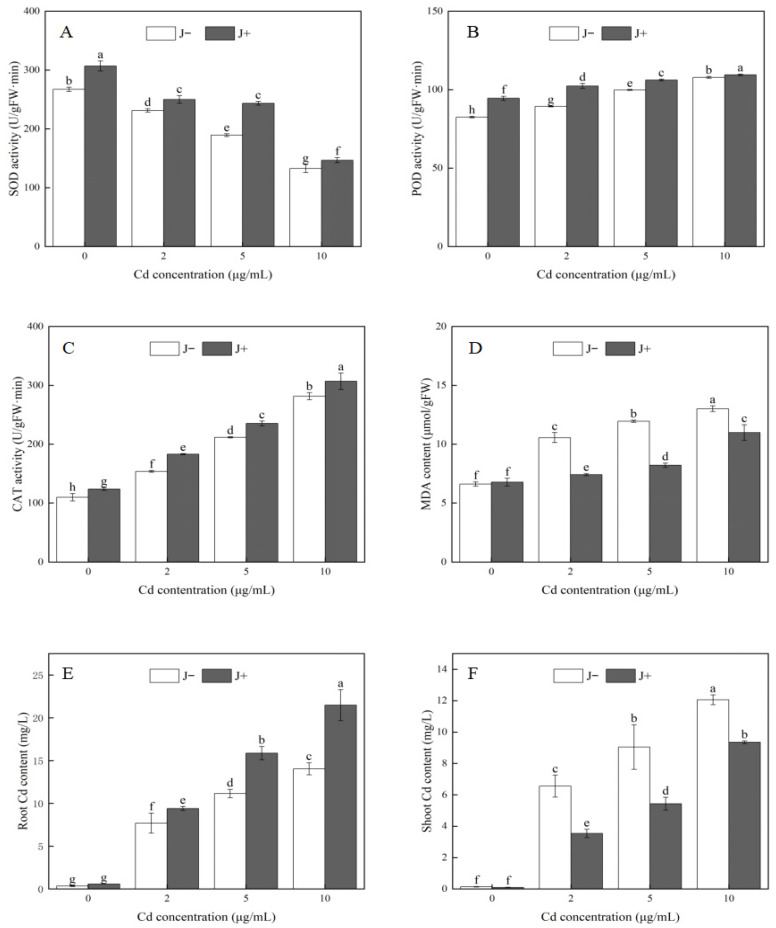
Strains KM25 (J+) and uninoculated (J−) of (**A**) (SOD), (**B**) (POD), (**C**) (CAT), (**D**) (MDA), (**E**) (root Cd content), and (**F**) (shoot Cd content). Each data point is the mean of three replicates. Error bars represent standard errors. Different letters indicate significant differences at *p* < 0.05.

To further explore the mechanisms underlying the growth-promoting effect of strain KM25 under Cd stress, we analyzed key physiological indicators related to oxidative stress in soybean seedlings with and without strain KM25 inoculation. In both J− and J+ soybean seedlings, SOD activity decreased as the Cd concentration increased (Figure 3A). Under different Cd concentration treatments, the SOD activity in the J+ seedlings was significantly elevated compared to the J− seedlings, increasing by 14.64%, 8.22%, 28.57%, and 10.61%, respectively (Figure 3A). With the increase in Cd concentration, the activities of POD and CAT in soybean seedlings showed an upward trend. Under Cd stress conditions, the activities of POD and CAT were higher in J+ seedlings compared to J− seedlings (Figure 3B,C). Specifically, the POD activity in the J+ group was significantly enhanced by 14.55%, 14.55%, 6.35%, and 1.55%, respectively, compared to the J− seedlings; the CAT activity was significantly increased by 12.77%, 19.07%, 11.14%, and 9.05%, respectively (Figure 3B,C). At all concentrations except 0 μg/mL, the MDA content showed a gradual increase with higher Cd concentrations, with significant differences observed (Figure 3D). Under Cd stress conditions, the MDA content in J+ seedlings was markedly lower than in J− seedlings, with reductions of 29.80%, 31.25%, and 15.57%, respectively (Figure 3D).

### 2.5. Cd Content

Beyond the oxidative stress responses, we also examined the accumulation and distribution of Cd in different parts of soybean seedlings to further clarify the role of strain KM25 in Cd stress alleviation. As Cd concentration increased, the Cd content in both the root and shoot portions of soybean seedlings showed a gradual upward trend (Figure 3D,E). However, at Cd concentrations of 2 μg/mL, 5 μg/mL, and 10 μg/mL, the Cd content in the roots of J+ seedlings increased by 22.23%, 42.32%, and 53.01%, respectively, compared to J− seedlings. In contrast, the Cd content in the shoot portions decreased by 46.01%, 39.99%, and 22.48%, respectively.

### 2.6. Diversity of Endophytic Bacterial Communities

**Figure 4:**
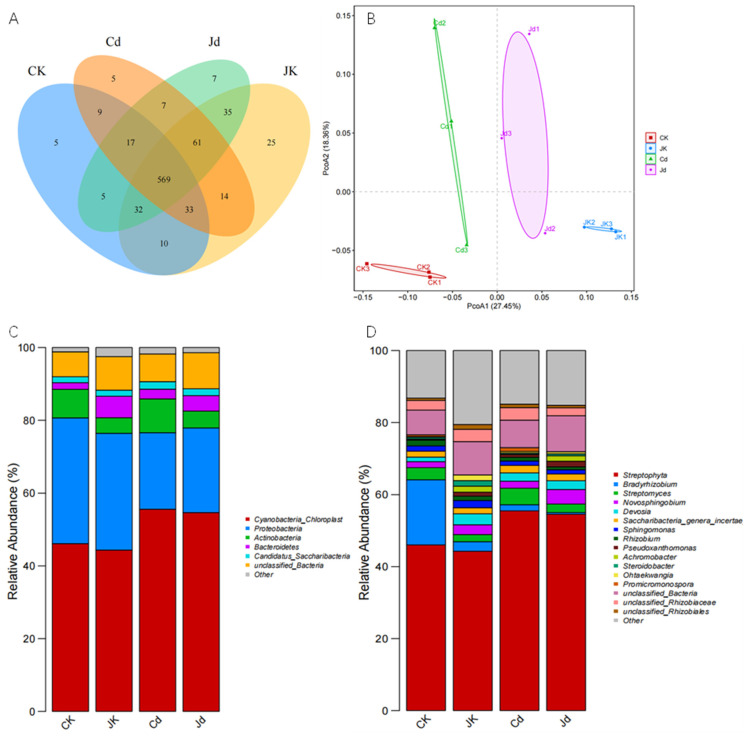
(**A**) Venn diagram; (**B**) the Bray–Curtis-based principal coordinate analysis (PCoA); the relative abundance of endophytic bacterial communities in the roots of soybean seedlings at the (**C**) phylum level and (**D**) genus level among different treatments.

Given that strain KM25 may influence Cd accumulation and plant responses through interactions with the endophytic microbiota, we further analyzed the structure of the endophytic bacterial community in soybean roots under different treatments. At a similarity of 97%, the sequences were clustered into OTUs (Operational Taxonomic Units), as shown in (Figure 4A). CK and Cd represent seedlings treated with Cd solutions at concentrations of 0 μg/mL and 10 μg/mL, respectively. JK and Jd represent seedlings treated with 0 μg/mL + 25 mL of KM25 fermentation broth (OD_600_ = 1, approximately 1 × 10^9^ CFU/mL, accounting for 5%) and 10 μg/mL Cd solution + 25 mL of LB liquid medium, respectively. A total of 834 OTUs were obtained through cluster analysis, with CK, Cd, Jd, and JK having 680, 715, 779, and 733 OTUs, respectively. Among them, 569 core OTUs were included. The four different treatment groups contained 5, 5, 25, and 7 unique OTUs, respectively. In the four different treatments, no significant differences were observed in the distribution richness (Chao, Ace), community diversity (Shannon, Simpson), and evenness (Shannon even) of the endophytic bacterial communities in soybean roots (Table 3). The Bray–Curtis-based principal coordinate analysis (PCoA) was conducted on the endophytic bacterial communities in roots (Figure 4B). The results indicated that PCoA1 and PCoA2 explained 27.45% and 18.36% of the variation in the endophytic bacterial communities in roots, respectively. There were significant differences among the endophytic bacterial communities in the four different treatments (Figure 4B).

### 2.7. Taxonomic Composition of Endophytic Bacterial Communities

To gain deeper insights into the taxonomic composition of the endophytic bacterial communities, we further analyzed their distribution at both the phylum and genus levels. To characterize the endophytic bacterial community composition at the phylum level, the top five taxa with the highest relative abundance were analyzed. These dominant phyla included Cyanobacteria, Proteobacteria, Actinobacteria, Bacteroidetes, and Candidatus_Saccharibacteria. Among them, Cyanobacteria exhibited the highest relative abundance (44.36–55.59%), representing the most dominant phylum (Figure 4C). Under normal conditions, the relative abundances of Cyanobacteria, Proteobacteria, and Actinobacteria in the JK group were lower than those in the CK group, whereas the relative abundances of Bacteroidetes and Candidatus_Saccharibacteria were elevated. Cadmium (Cd) stress induced a 9.46% increase in the relative abundance of Cyanobacteria but a 13.51% decrease in that of Proteobacteria. Compared with the Cd-treated group, the Jd group showed increased relative abundances of Proteobacteria (by 2.22%) and Bacteroidetes (by 1.57%), accompanied by a 4.64% reduction in the relative abundance of Actinobacteria. At the genus level, the top 10 bacterial genera were *Streptophyta*, *Bradyrhizobium*, *Streptomyces*, *Novosphingobium*, *Devosia*, *Saccharibacteria gera incertae_sedis*, *Sphingomonas*, *Rhizobium*, *Pseudoxanthomonas*, and *Achromobacter* (Figure 4D). The dominant genus of endophytic bacteria in soybean roots was *Streptophyta* (44.28–55.50%). Compared with the CK group, the abundances of *Novosphingobium*, *Devosia*, *Rhizobium*, *Pseudoxanthomonas*, and *Achromobacter* in the Jd group increased, while that of *Bradyrhizobium* decreased by 15.45%. Compared with the Cd group, the abundances of *Novosphingobium*, *Rhizobium*, and *Pseudoxanthomonas* in the Jd group increased by 2.11%, 0.81%, and 1.38%, respectively, while the abundance of *Bradyrhizobium* decreased.

### 2.8. Functional Prediction Analysis of Endogenous Bacterial Communities

**Figure 5:**
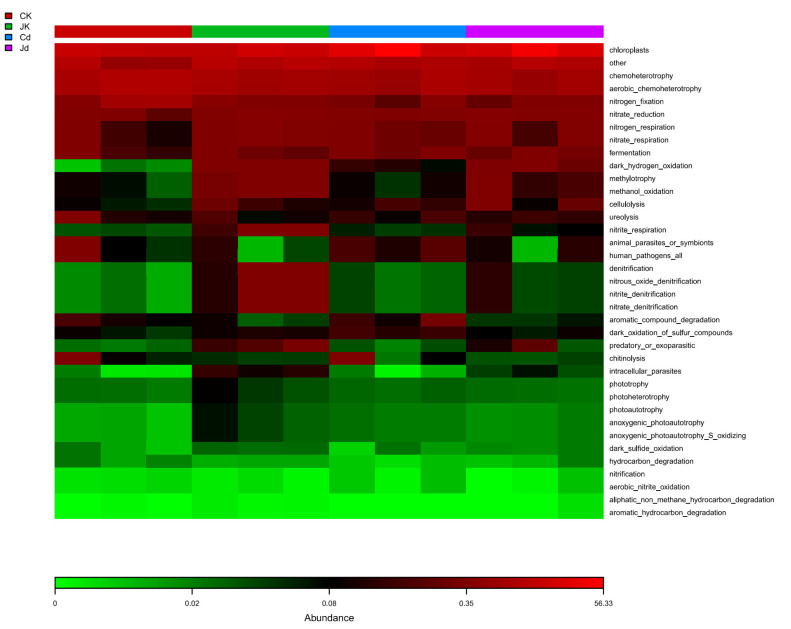
Functional differences in endophytic bacterial communities in soybean roots under different treatments were analyzed using FAPROTAX.

The functional differences in endophytic bacteria in the roots of soybean plants under different treatments were compared using the database FAPROTAX. Compared with the CK and Cd groups, after inoculation with strain KM25, the relative abundance of bacteria with functions such as nitrate reduction, nitrogen respiration, nitrate respiration, dark hydrogen oxidation, methanol oxidation, and aromatic hydrocarbon degradation increased (Figure 5).

## 3. Discussion

In this study, a *Pseudomonas* sp. strain KM25 was isolated and screened. Its plant growth-promoting (PGP) characteristics, alleviating effect on soybean seedlings under Cd stress, and impact of its inoculation on the endophytic bacterial community in soybean roots were investigated and analyzed.

The plant hormone IAA is essential for stimulating growth and improving the absorption of nutrients in plants [30]. Siderophores, a group of highly specific iron-chelating compounds secreted by bacteria, improve the bioavailability of iron in plants under stress conditions, thereby supporting and optimizing plant development [31]. Additionally, ACC deaminase enzymatically hydrolyzes ACC, reducing ethylene accumulation in roots and mitigating the adverse effects of heavy metals on plant growth and development [32]. In this study, strain KM25 was found to produce IAA and siderophores, solubilize organic and inorganic phosphorus, and exhibit ACC deaminase activity. Similarly, *B. cereus* WSE01, MEN8, YL6, and SA1 have shown a number of significant growth-promoting traits, while IAA and ACC deaminase production or phosphate dissolution [33].

Cd, a non-essential and highly toxic heavy metal, has the ability to accumulate in significant amounts within plants. This accumulation disrupts the physiological and biochemical processes occurring in plant cells and suppresses growth [34]. In this research, various levels of Cd stress significantly inhibited the growth of soybean seedlings (Table 2). Under high concentration Cd stress, the growth indicators of soybean seedlings, such as plant height, root length, dry weight, fresh weight, and chlorophyll content, were all significantly reduced, which visually reflected the severe inhibition of Cd on the growth of soybean seedlings. However, after adding strain KM25, this inhibitory effect was significantly alleviated, indicating that strain KM25 has a positive effect on the response of soybean seedlings to Cd stress (Table 2). The pot experiment results by Patel et al. showed that under Cd stress, strain *Curtobacterium oceanosedimentum* DG-20 could promote the growth of peppers. Compared with the control, the root length and stem length of peppers increased by 58% and 60%, respectively, after inoculation with this strain, and the fresh weight and dry weight also increased [35]. The pot experiment showed that compared with the uninoculated control, inoculation with *Bacillus* QX8 and QX13 significantly increased the stem and root dry weight of plants in Cd- and Pb-contaminated soil (1.36 times and 1.7 times, and 1.42 times and 1.96 times, respectively) [7]. Strain KM25 can stimulate the growth and development of roots, which not only benefits plants in better absorbing water and nutrients but also may enhance their survival ability in Cd stress environments. This indicates that strain KM25 plays an important role in promoting the growth of both the shoot and root parts of soybean seedlings.

Cd stress triggers the buildup of reactive oxygen species (ROS) in plants, causing oxidative harm to proteins, lipids, and DNA [36]. MDA, which is produced during membrane lipid peroxidation, has been utilized as an indicator to assess the degree of oxidative damage to lipids in plants [37]. Plants will produce POD, SOD, CAT, and other antioxidant enzymes to remove excess ROS and enhance plant resistance to Cd [38]. In this research, the MDA content in the inoculated strain KM25 was found to be lower compared to the uninoculated group under different Cd concentrations (Figure 3D). As a major product of lipid peroxidation, lower levels of MDA imply less damage to cell membranes. This result suggests that strain KM25 might regulate the physiological condition of the cell membrane, thereby lessening the harmful effects of Cd on the membrane system and maintaining the integrity and normal functionality of the cell. The result was similar to those of previous studies. The research by Wei et al. demonstrated that, under Cd stress, inoculation with *Serratia* sp. D23 and *Sphingomonas* sp. D36 can enhance the levels of photosynthetic pigments in tomato leaves while decreasing the MDA content in tomato roots [39]. This study revealed that SOD activity progressively declined as Cd concentration increased, whereas POD and CAT activities showed a gradual increase (Figure 3A–C). Under all Cd treatments, SOD, POD, and CAT activities were higher in the J+ group than in the J− group. Wu et al. discovered that *Serratia marcescens* SNB6 increased the activity and detoxification ability of antioxidant enzymes in *C. zizanioides*, thereby enhancing its tolerance to Cd. [40]. The bacterial isolate *Bacillus cereus* ALT1, when used for inoculation, decreased the abscisic acid (ABA) content, boosted salicylic acid (SA) production, and stimulated antioxidant responses through an increase in total protein (TP) and SOD levels [5]. Inoculation with the rhizobacteria isolate *Pseudocitrobacter anthropic* C18 enhanced the production of antioxidant enzymes, including CAT, ascorbate oxidase (ASO), and POD activities, while also decreasing the accumulation of ROS in leaves [41]. Strain KM25 might enhance the antioxidant defense system of soybean seedlings by increasing the activities of SOD, POD, and CAT, which in turn lessens the cellular damage resulting from Cd stress.

This study investigated the impact of strain KM25 on Cd absorption and transportation in soybean seedlings under varying degrees of Cd stress. The results demonstrated that the patterns of Cd accumulation in the root and shoot of soybean plants exhibited significant differences, and strain KM25 played a substantial role in these processes (Figure 3E,F). In this study, it was observed that the Cd content in the roots of soybean plants was significantly higher than in the shoots for both J+ and J− seedlings. This finding is consistent with a previous study that reported higher Cd accumulation in roots compared to stems and leaves under Cd stress [42]. As the organs directly exposed to Cd in the soil, roots are crucial in preventing the excessive upward transport of Cd [43]. Under medium–high Cd stress, the Cd content in the roots of J+ seedlings increased significantly compared with that in J− seedlings (Figure 3E). On the contrary, the Cd content in the shoot part of the J+ seedlings was significantly reduced, which indicated that strain KM25 enhanced the Cd uptake capacity of roots and limited the transfer of Cd from roots to shoots part (Figure 3F). PGPR can alter the morphology of heavy metals in soil by secreting organic acids and other substances, enhancing their solubility and availability. This process enhances the absorption of heavy metals by the plant’s root system, leading to a greater accumulation of heavy metals in the roots [44].

In a Cd-contaminated environment, the structure and activity of microbial communities are significantly altered [45]. In this study, the inoculation of strain KM25 did not significantly affect the α-diversity of the endophytic bacterial community in soybean roots. Nevertheless, the PCoA results demonstrated that the structure of the endophytic bacterial community in the rhizosphere was altered (Figure 4A,B). Following inoculation with strain KM25, the relative abundance of Proteobacteria increased at the phylum level (Figure 4C). Similarly, Zheng et al. reported that inoculating *Serratia marcescens* WZ14 modified the composition of the rhizosphere bacterial communities in two leguminous plants, leading to an increase in the relative abundance of Proteobacteria and Firmicutes [46]. Several studies have shown that Proteobacteria exhibit high Cd tolerance and are commonly found in soils contaminated with heavy metals [47]. Proteobacteria are capable of converting nitrogen gas in the air into a form accessible to plants, providing nitrogenous nutrition for plants and facilitating their growth [48]. Furthermore, after inoculation with strain KM25, the relative abundance of the Bacteroidetes phylum also witnessed an increase. The Bacteroidetes phylum is involved in the cycling of carbon, nitrogen, and phosphorus, contributes to various metabolic pathways, and plays a crucial role in soil nutrient cycling [49]. Hence, strain KM25 might promote the metabolic activities of soybean seedlings and alleviate the harm brought by Cd by recruiting beneficial Proteobacteria and Bacteroidetes in the soil. At the genus level, this study revealed an increase in the relative abundances of the genera *Sphingomonas*, *Rhizobium*, and *Pseudoxanthomonas* within the Proteobacteria phylum following inoculation with strain KM25 (Figure 4D). Under the combined stress of NaCl and CdCl_2_, *Rhizobial* inoculation significantly reduces catalase activity and inhibits cadmium uptake in the roots of Alfalfa. Furthermore, gene expression analysis demonstrated that *Rhizobial* inoculation enhanced the biosynthesis of proline and phytochelatins, which may contribute to alleviating oxidative stress and detoxifying heavy metals [50]. The bacterial genus *Novosphingobium* exhibited a range of ecological functions, including the degradation of pesticides, resistance to heavy metals, capacity for nitrogen fixation, antibiotic resistance, and participation in sulfur metabolic processes [51]. The Pseudomonas strain YP1 has been verified to possess the capability for denitrifying phosphorus removal (DPR) [52]. Therefore, the inoculation of strain KM25 enriched *Sphingomonas*, *Rhizobium*, and *Pseudoxanthomonas*, promoting the growth of soybeans. Following inoculation with strain KM25, the relative abundance of bacteria involved in processes such as nitrate reduction, denitrification, nitrate respiration, dark hydrogen oxidation, methanol oxidation, and aromatic hydrocarbon degradation was observed to increase (Figure 5). Chemical energy, aerobic chemolithotrophy, and fermentation are important ecological functions related to the carbon cycle [53]. This indicates that the inoculated strain may have promoted the nitrogen cycle in soybean seedlings and helped release many inorganic nutrients that the host can utilize from organic matter.

## 4. Materials and Methods

### 4.1. Isolation and Identification of Strain KM25

Root nodules samples were collected from the experimental field at Shenyang Normal University (41.908781° N, 123.41526° E), Shenyang Liaoning Province, China. Samples were collected in sterile bags, transported to the lab in ice boxes, and subsequently stored at 4 °C. The surface sterilization procedure for root nodules samples was as follows: First, rinse with deionized water 3 times, followed by treatment with 75% ethanol for 40 s, and then successive treatment with 2% NaClO twice, 40 s each time. The samples were finally rinsed 5 times with deionized water. To confirm the success of the surface sterilization, the deionized water from the final rinse was spread onto Ashby’s agar plates (per liter of distilled water: mannitol 10.0 g, KH_2_PO_4_ 0.2 g, MgSO_4_·7H_2_O 0.2 g, NaCl 0.2 g, CaCO_3_ 5.0 g, and agar 20.0 g) and incubated at 37 °C for 48 h to ensure no microbial growth occurred. The surface-sterilized root nodules are placed in a sterilized mortar and ground with sterile water. A 100 μL aliquot of the homogenized root nodule samples was spread onto Ashby’s agar plates and incubated at 37 °C for 48 h to screen and obtain endophytic PGPR. The single colonies were purified by employing the streak plate method, and the purified strains were preserved at −80 °C mixed with 25% glycerol.

### 4.2. Screening Cadmium-Tolerant Strains

The 12 isolated PGPR strains were inoculated into Luria–Bertani (LB) liquid medium (per liter of distilled water: Tryptone: 10.0 g, Yeast extract: 5.0 g, NaCl: 10.0 g) containing varying concentrations of CdCl_2_ (0, 50, 150, 250, and 300 μg/mL) at an inoculation rate of 1%. They were then cultured at 37°C and 160 rpm for 24 h. The optical density at 600 nm (OD_600_) was determined using a UV–visible spectrophotometer (Model T-UV1810, Shanghai Youke Instrument Co., Ltd., Shanghai, China).

### 4.3. DNA Extraction and Bacterial Identification

The genomic DNA of the strain KM25 was extracted by the colony cracking method. The universal primers 27F (5′-AGAGTTTGATCCTGGCTCAG-3′) and 1492R (5′-GGTTACCTTGTTACGACTT-3′) were used to amplify the 16S rRNA gene from the extracted DNA. The PCR products were purified and sequenced by Sangon Biotech (Shanghai, China). The sequence of strain KM25 was analyzed by performing BLAST + 2.9.0 alignment and classification in GenBank via the National Center for Biotechnology Information (NCBI) database. A phylogenetic tree was then generated using the neighbor-joining method implemented in MEGA 11.0 software [54].

### 4.4. Determination of PGP Traits of PGPR

The activated KM25 strain was inoculated into PKO liquid medium (glucose 10.0 g, ammonium sulfate 0.5 g, sodium chloride 0.3 g, calcium phosphate 2.0 g, magnesium sulfate heptahydrate 0.3 g, potassium chloride 0.3 g, ferrous sulfate heptahydrate 0.036 g, manganese sulfate monohydrate 0.03 g, water 1000 mL), organophosphorus yolk liquid medium (glucose 10 g, ammonium sulfate 0.5 g, magnesium sulfate heptahydrate 0.3 g, sodium chloride 0.3 g, potassium chloride 0.3 g, ferrous sulfate heptahydrate 0.03 g, manganese sulfate monohydrate 0.03 g, calcium carbonate 5 g, sterile egg yolk 50 mL, distilled water 1000 mL, pH 7.0–7.5), SA medium (per liter of distilled water: casein peptone 5 g, beef extract 5 g, chromogene mixture 5.81 g, Agar 12.8 g), King medium (peptone 20 g, K_2_HPO_4_ 1.725 g, MgSO_4_⋅7H_2_O 1.5 g, glycerol 15 mL, tryptophan 0.1 g, distilled water 1000 mL), and LB medium at a 1% inoculation volume, respectively. The cultures were incubated at 37 °C with shaking at 160 rpm for 7 d and subsequently centrifuged at 8000 rpm for 15 min. The phosphorus-solubilizing capacity of the strain was assessed using the molybdenum antimony colorimetric method [55]. The siderophore production activity of the strain was evaluated using CAS staining and light absorption method [56]. The concentration of IAA produced by the strain was measured using the Salkowski colorimetric method [57]. The ACC deaminase activity of the strain was measured using the 2,4-dinitrophenylhydrazine colorimetric method [58].

### 4.5. Hydroponic Experiment

Healthy and uniformly sized soybean seeds were selected for surface sterilization: First, they were soaked in 75% ethanol for 40 s, and then soaked in 2% sodium hypochlorite (NaClO) for 3 min. After being washed with deionized water 5–6 times, the seeds were incubated in the dark at 28 °C until germination. Seeds with buds of 2–3 cm in length were selected and transferred to hydroponic boxes of 1/4 Hoagland nutrient solution, and were grown in an artificial climatic cabinet under conditions of 28 °C, 300 μM·m^−2^·s^−1^ light intensity, 80% air humidity, and 16 h/8 h light/dark photoperiod. Seedlings were transplanted into 1/4 Hoagland nutrient solution with varying Cd concentrations (0, 2, 5, 10 μg/mL). Approximately 5% fermentation broth of strain KM25 was inoculated to seedlings. The bacterial and Cd treatment groups were named Jd0, Jd2, Jd5, and Jd10 (J+), and the control groups with added NB medium were named Cd0, Cd2, Cd5, and Cd10 (J−). Three parallel experiments were set for each group. After 14 d, the seedlings were sampled and the physio-biochemical indicators including shoot and root biomass and chlorophyll content, the activity of antioxidant enzymes SOD, CAT, and peroxidase (POD), and the content of MDA were all measured.

#### 4.5.1. Determination of Biomass and Chlorophyll Content of Soybean Seedlings

Complete soybean seedlings were collected and divided into shoot and root parts. The shoot height, root length, fresh weight, and dry weight after drying to a constant weight were measured. The chlorophyll content of the leaves was determined using a portable chlorophyll meter (Model YT-YA, Shandong Yuntang Intelligent Technology Co., Ltd., Weifang, China).

#### 4.5.2. Determination of Antioxidant Enzymes and Lipid Peroxidation

Five grams of the sample were carefully weighed and subsequently placed into a mortar. Five milliliters of phosphate-buffer solution were added, and the mixture was subsequently homogenized under ice-cold conditions. The homogenate was centrifuged at 4 °C and 12,000× *g* for 30 min to collect the supernatant. The activities of SOD, POD, and CAT as well as the content of MDA were determined by the nitroblue tetrazolium (NBT) colorimetric method, guaiacol spectrophotometric method, hydrogen peroxide decomposition method, and thiobarbituric acid reactive substances method, respectively [59].

#### 4.5.3. Determination of Cd Content

The shoots and roots of soybean seedlings were separated. The samples were rinsed with distilled water and oven-dried for 30 min at 105 °C, then at 80 °C until constant weight. Samples were digested with HNO_3_ and H_2_O_2_ and concentrations of Cd in the digests were determined by inductively coupled plasma mass spectrometry (ICP-MS) [60].

### 4.6. Analysis of the Community Structure of Endophytic Bacteria in Soybean Roots

According to the germination method described in Section 2.5, soybean seeds were planted in pots filled with a mixture of peat soil and vermiculite (1:1). The experiment was divided into two groups: (1) treated with 0 μg/mL and 10 μg/mL concentrations of Cd solution, labeled as control (CK) and Cd, respectively; (2) treated with 0 μg/mL and 10 μg/mL concentrations of Cd solution + KM25 fermentation liquid, marked as JK and Jd, respectively. Each group had three replicates. During the seedling cultivation period, the pots were constantly exchanged in position to ensure that the nutritional conditions and growth environment of the plants were basically the same. After the potted culture entered the mature stage, root samples of soybeans under different treatments (CK, Cd, JK, Jd) were collected.

#### 4.6.1. Samples Treatment

After the pot experiment entered the mature stage, root samples of soybeans under different treatments (CK, Cd, JK, Jd) were collected. High-throughput sequencing technology was used to analyze the effect of strain KM25 on the community structure of endophytic bacteria in soybean roots under Cd stress. Each treatment had three replicates, and the samples were sent to Sangon Biotech (Shanghai) Co., Ltd. for sequencing.

#### 4.6.2. DNA Extraction

DNA was extracted using the OMEGA E.Z.N.ATM Mag-Bind Soil DNA Kit (Omega Bio-tek, Norcross, GA, USA).

#### 4.6.3. PCR Amplification

The genomic DNA was accurately quantified using the Qubit 3.0 DNA Assay Kit. PCR amplification of the 16S rRNA gene was performed using primers 341F (5′-CCTACGGGNGGCWGCAG-3′) and 805R (5′-GACTACHVGGTATCTAATCC-3′). The reaction mixture was prepared in a sterile PCR tube as shown in Table 4. Illumina bridge PCR-compatible primers were introduced for the second round of amplification, with the reaction system detailed in Table 4. Thermal cycling conditions were as follows: initial denaturation at 95 °C for 5 min; followed by 25 cycles of denaturation at 95 °C for 30 s, annealing at 50 °C for 30 s, and extension at 72 °C for 40 s; and a final extension at 72 °C for 7 min.

#### 4.6.4. High-Throughput Sequencing of the V3–V4 Region of Bacterial 16S rDNA

The DNA samples obtained by PCR amplification were subjected to amplification of the V3–V4 region of the 16S rDNA, followed by high-throughput sequencing. The resulting data were then analyzed. The functional roles of strain KM25 in sulfur, carbon, hydrogen, and nitrogen cycling were predicted using the FAPROTAX database [61].

### 4.7. Data Processing and Analysis

One-way ANOVA and LSD multiple range tests were carried out with SPSS 25.0 to evaluate the systematic differences among treatments and between the two groups, respectively (*p* < 0.05). All experiments were carried out in triplicate. Data are presented as mean ± standard deviation (SD). Origin 2021 was used for graphical presentation. OTU clustering analysis was performed using USEARCH v11.0 software. High-quality sequences were clustered into OTUs at a 97% sequence similarity threshold, and chimeras were removed using the UCHIME algorithm. The most abundant sequence of each OTU was selected as the representative sequence, which was then aligned against the Silva v138 database (using BLASTn algorithm (version 2.14.0+) with a minimum similarity of 80%) for taxonomic annotation. Based on the OTU abundance matrix, Bray–Curtis distances were calculated to quantify community dissimilarities among samples, and non-metric multidimensional scaling (NMDS) and principal coordinate analysis (PCoA) were performed to visualize separation patterns between groups. Significance of intergroup differences was tested using ANOSIM and Adonis (PERMANOVA) with 999 permutations and evaluated by *R* and *p* values (*p* < 0.05 indicating significance). At phylum/genus levels, the top 10 taxa by relative abundance were visualized via bar charts, with rare taxa (<1%) grouped as “Others”. Differential abundance was tested using Kruskal–Wallis (multi-group) or Wilcoxon (two-group) tests, followed by the FDR correction (Benjamini–Hochberg method, adjusted *p* < 0.05). Key biomarkers were identified via LEfSe (LDA > 4), and effect sizes were quantified by Log_2_FC.

## 5. Conclusions

In this research, strain KM25 with high Cd tolerance was isolated and screened from the root nodules of semi-wild soybean. The results indicated that strain KM25 was capable of generating IAA, siderophores and ACC deaminase, as well as dissolving both organic and inorganic phosphorus. This strain not only enhanced the growth parameters and antioxidant activities of soybean seedlings under Cd stress, but also decreased the MDA content, increased Cd absorption in the roots of soybean seedlings, and reduced the Cd content in the shoot parts. Furthermore, strain KM25 demonstrated the ability to modulate the structure of the endophytic bacterial community within soybean roots, while also acclimating to and mitigating the harm induced by Cd.

## Figures and Tables

**Table 1:** OD_600_ values of bacterial strains grown under different CdCl_2_ concentrations.

Strain	CdCl_2_ Concentration (μg/mL)				
	0	50	150	250	300
KM01	0.827 ± 0.054 a	0.449 ± 0.077 b	0.151 ± 0.041 c	0.091 ± 0.006 c	0.051 ± 0.017 c
KM04	0.661 ± 0.020 a	0.390 ± 0.036 b	0.141 ± 0.050 c	0.074 ± 0.004 d	0.035 ± 0.020 d
KM06	0.792 ± 0.072 b	0.884 ± 0.005 a	0.411 ± 0.049 c	0.114 ± 0.022 d	0.097 ± 0.006 d
KM10	0.739 ± 0.011 a	0.627 ± 0.055 b	0.220 ± 0.062 c	0.086 ± 0.009 d	0.058 ± 0.027 d
KM14	0.859 ± 0.073 a	0.640 ± 0.126 b	0.296 ± 0.101 c	0.082 ± 0.007 d	0.067 ± 0.009 d
KM15	0.744 ± 0.036 a	0.625 ± 0.026 b	0.308 ± 0.030 c	0.076 ± 0.006 d	0.057 ± 0.018 d
KM18	0.746 ± 0.044 a	0.849 ± 0.027 b	0.386 ± 0.097 c	0.076 ± 0.006 d	0.078 ± 0.020 d
KM19	0.793 ± 0.066 a	0.673 ± 0.026 b	0.323 ± 0.028 c	0.090 ± 0.001 d	0.075 ± 0.015 d
KM25	0.754 ± 0.025 b	0.926 ± 0.034 a	0.692 ± 0.039 c	0.376 ± 0.041 d	0.248 ± 0.044 e
KM32	0.754 ± 0.022 b	0.842 ± 0.017 a	0.469 ± 0.051 c	0.205 ± 0.007 d	0.121 ± 0.060 e
KM34	0.745 ± 0.009 b	0.825 ± 0.014 a	0.486 ± 0.033 c	0.129 ± 0.035 d	0.128 ± 0.054 d
KM38	0.775 ± 0.021 a	0.645 ± 0.124 b	0.085 ± 0.001 c	0.078 ± 0.001 c	0.069 ± 0.006 c

Values indicate the means ± standard deviation (*n* = 3). In each column, values labeled with different letters are significantly different (at *p* < 0.05).

**Table 2:** Biomass and chlorophyll content of soybeans under different treatments.

	SH (cm)	RL (cm)	SFW (g)	SDW (g)	RFW (g)	RDW (g)	CC (SPAD)
Cd0	16.9 ± 1.237 bc	12.67 ± 0.15 b	3.22 ± 0.05 b	1.04 ± 0.02 b	3.31 ± 0.06 b	1.66 ± 0.1 b	34.47 ± 0.15 b
Cd2	14.32 ± 1.2 de	15.5 ± 0.2 a	2.52 ± 0.12 d	0.63 ± 0.01 c	2.6 ± 0.09 d	1.27 ± 0.03 cd	32.23 ± 1.09 c
Cd5	12.84 ± 0.563 e	10.67 ± 0.21 c	1.79 ± 0.06 e	0.54 ± 0.01 cd	2.43 ± 0.02 e	0.93 ± 0.04 e	29.43 ± 0.15 e
Cd10	9.86 ± 0.736 f	9.3 ± 0.61 d	1.33 ± 0.07 f	0.31 ± 0.01 d	2.07 ± 0.06 f	0.74 ± 0.11 e	28.87 ± 0.87 e
Jd0	21.26 ± 2.018 a	10.47 ± 0.31 c	3.48 ± 0.03 a	1.47 ± 0.35 a	3.51 ± 0.06 a	1.97 ± 0.17 a	38.23 ± 0.21 a
Jd2	17.68 ± 1.003 b	12.37 ± 0.12 b	2.78 ± 0.08 c	0.69 ± 0.01 c	2.74 ± 0.02 c	1.39 ± 0.11 c	34.33 ± 1.01 b
Jd5	15.15 ± 1.089 cd	9.27 ± 0.15 d	2.39 ± 0.28 d	0.61 ± 0.01 c	2.58 ± 0.01 d	1.15 ± 0.08 d	30.87 ± 0.61 d
Jd10	12.485 ± 1.903 e	6.57 ± 1.81 e	1.63 ± 0.13 e	0.35 ± 0.01 d	2.46 ± 0.06 e	0.82 ± 0.06 e	29.4 ± 0.36 e

Values represent means ± standard deviation (*n* = 3). Values marked with different letters in each column are significantly different (at *p* < 0.05). SH (shoot height), RL (root length), SFW (shoot fresh weight), SDW (shoot dry weight), RFW (root fresh weight), RDW (root dry weight), CC (chlorophyll content).

**Table 3:** Alpha diversity index.

	Shannon	Chao	Ace	Simpson	Shannon even
CK	2.47 ± 0.47 a	633.72 ± 25.15 a	636.78 ± 30.55 a	0.27 ± 0.05 a	0.39 ± 0.07 a
Cd	2.56 ± 0.58 a	653.41 ± 52.66 a	648.79 ± 49.26 a	0.32 ± 0.11 a	0.40 ± 0.08 a
JK	3.18 ± 0.36 a	718.19 ± 23.27 a	724.25 ± 14.63 a	0.21 ± 0.05 a	0.49 ± 0.01 a
Jd	2.53 ± 0.26 a	675.02 ± 46.27 a	665.62 ± 42.93 a	0.31 ± 0.06 a	0.41 ± 0.04 a

Values represent means ± standard deviation (*n* = 3). Different letters indicate significant differences at *p* < 0.05.

**Table 4:** Reaction system.

Component	Volume
2×Hieff^®^ Robust PCR Master Mix	15 µL
10 μM Primer R	1 µL
Bar-PCR primer F	1 µL
ddH_2_O	9~12 µL
PCR products	10~20 ng
Total volume	30 μL

## Data Availability

The original contributions presented in this study are included in the article. Further inquiries can be directed to the corresponding authors.
